# Guideline-Optimised Treatment in Heart Failure—Do Higher Doses Reduce Systemic Inflammation More Significantly?

**DOI:** 10.3390/jcm13113056

**Published:** 2024-05-23

**Authors:** Alexandru Mircea Arvunescu, Ruxandra Florentina Ionescu, Silviu Ionel Dumitrescu, Ondin Zaharia, Tiberiu Ioan Nanea

**Affiliations:** 1 Department of Cardio-Thoracic Pathology, Faculty of Medicine, Carol Davila University of Medicine and Pharmacy, 050471 Bucharest, Romania; ondin.zaharia@umfcd.ro (O.Z.); tiberiu.nanea@yahoo.com (T.I.N.); 2 Department of Internal Medicine and Cardiology, “Prof. Dr. Th. Burghele” Clinical Hospital, 061344 Bucharest, Romania; 3 Department of Cardiology I, Central Military Emergency Hospital “Dr. Carol Davila”, 030167 Bucharest, Romania; dr.ruxandra.ionescu@gmail.com (R.F.I.); dr.silviu.dumitrescu@gmail.com (S.I.D.); 4 Department of Cardiology, Faculty of Medicine, Titu Maiorescu University, 040441 Bucharest, Romania

**Keywords:** heart failure, inflammation, CRP, ESR, fibrinogen, beta-blockers, ACE-I, ARB, MRA, SGLT2i, ARNI, left ventricular ejection fraction

## Abstract

**Background:** Chronic inflammation is a constant phenomenon which accompanies the heart failure pathophysiology. In all phenotypes of heart failure, irrespective of the ejection fraction, there is a permanent low-grade activation and synthesis of proinflammatory cytokines. Many classes of anti-remodelling medication used in the treatment of chronic heart failure have been postulated to have an anti-inflammatory effect. **Methods:** This retrospective study enrolled 220 patients and focused on evaluating the effect of the most used active substances from these classes in reducing the level of inflammatory biomarkers (C reactive protein, erythrocyte sedimentation rate and fibrinogen) after initiation or up-titration. Our research is evaluating if this anti-inflammatory effect intensifies while raising the dose. The evaluation was performed at two visits with an interval between them of 6 months. **Results:** From the beta-blockers class, carvedilol showed a reduction in erythrocyte sedimentation rate (ESR), in low (6.25 mg, bi daily) and medium (12.5 mg, bi daily) doses. At the same time, sacubitril/valsartan showed a reduction in CRP levels. This effect was obtained only in the medium (49/51 mg, bi daily) and high (97/103 mg, bi daily) doses, with the maximum reduction being observed in the high dose. **Conclusions:** From the classes of medication evaluated, the study showed a significant reduction in ESR levels in the low and medium doses of carvedilol and a reduction in CRP values in the cases of medium and high doses of ARNI.

## 1. Introduction

Heart failure (HF) is a global burden of healthcare systems worldwide, unfortunately with high rates of mortality and morbidity. The central role of inflammation in the pathophysiology of HF disease progression is evidenced by elevated concentrations of inflammatory markers, alongside characteristic neurohormonal and sympathetic nervous system activation [1].

In HF, both the innate and the adaptive immune systems are activated, involving B and T cells, macrophages, and mast cells [2]. The reciprocal relationship between HF and inflammation has been intensely discussed for a long time.

Inflammation reflects the immune system’s response to harmful foreign stimuli. No matter the trigger, the inflammatory cascade comprises well established phases. In the early phase, there is an activation of circulating blood cells, such as leukocytes, mast cells, dendritic cells, and T naive lymphocytes produce different cytokines, such as IL-10, IL-6, Il-1 beta, and IL-18. The repair phase consists of the involvement of multicellular protein expression, specialised matrix protein and cellular activation (fibronectin, proteoglycans, cardiomyocytes, fibroblasts, macrophages, and vascular cells). Failure to perpetuate this latter phase leads to the impossibility to regain myocardial function, therefore promoting the development of HF [3].

Inflammation is involved in the pathophysiology of the entire spectrum of HF, irrespective of ejection fraction (EF) of the left ventricle (LV). No clinical consensus has been made, because the majority of trials failed to identify successful anti-inflammatory treatment strategies for HF patients. However, it is worth mentioning that CRP (C reactive protein), tumoral necrosis factor, IL-1, IL-6, IL-8, IL-10, IL-18, IL-33, myeloperoxidase and inducible nitric oxide synthase can be regarded as potential therapeutic targets [4].

In HFpEF, oxidative stress and inflammation are the first triggers of cardiomyocyte dysfunction. Inflammation generates microvascular dysfunction, modifying the interaction between coronary microcirculation and cardiomyocytes. Tissue samples revealed high endothelial reactive oxygen species production and low nitric oxide bioavailability for HFpEF [5].

In HFrEF, ischemia or toxicity often leads to ventricular remodelling after cardiomyocyte necrosis. Cardiomyocyte injury perpetuates the inflammatory response, leading to progressive LV remodelling and dysfunction [5].

HFrEF is associated with high circulating concentration of proinflammatory cytokines. The most evaluated marker of inflammation is CRP. One trial concludes that in patients with established cardiovascular disease, CRP was an independent risk factor for incident HF [6]. A sub analysis of Val-HeFT (valsartan in HF) trial indicated that high hsCRP (highly sensitive C reactive protein) concentrations are independently associated with mortality and morbidity, with incremental prognostic value to that provided by B type natriuretic peptide alone [7]. In patients with CHF (chronic heart failure), high hsCRP concentrations were associated with an accentuated status of congestion and a worse prognosis, irrespective of NTproBNP (N-terminal pro b-type natriuretic peptide) [8]. However, evidence is not consistent in what concerns hsCRP, since other studies did not find additional prognostic information for hsCRP over the already known outcome predictors for HF [5,9].

A study conducted by Park and colleagues pointed out that in patients with acute HF, in which the decompensation was not attributed to an infection, CRP was a prognostic marker for both HFrEF and HFpEF. Furthermore, patients with high CRP levels had beneficial outcomes after treatment with statins, a class proven to have also anti-inflammatory effects besides the already known mechanism of lowering LDL-cholesterol [6].

Elevated fibrinogen levels are associated with traditional cardiovascular risk factors and can be regarded as a strong primary risk factor for cardiovascular disease in healthy subjects [10,11,12]. Fibrinogenis associated with HF prognosis, predicting mortality in critically ill patients with acute exacerbation of CHF [13]. Also, fibrinogen can be associated with long-term all cause and cardiac mortality in patients suffering from coronary artery disease undergoing percutaneous interventions, especially in those with diabetes mellitus [14].

Erythrocyte sedimentation rate (ESR), another marker of systemic inflammation was correlated with disease severity in patients with chronic HF [15]. Moreover, in a study which enrolled subjects without HF, myocardial infarction or valvular disease ESR was a significant predictor of HF, independent of classical HF risk factors [16]. In chronic HF, ESR was a negative prognostic sign, independent of the symptomatology (NYHA class- New York Heart Association), EF or peak oxygen consumption [17]. ESR is a strong predictor of coronary heart disease mortality, being associated with severe forms of coronary heart disease [18].

It was suggested that prompt initiation of anti-inflammatory therapy possesses favourable effects on the heart [19].

Until CANTOS (Canakinumab Anti-inflammatory Thrombosis Outcome Study) which reached the results in 2019, most efforts of targeting inflammation in HF were not accompanied by great success. CANTOS trial evaluated anti-cytokine treatment with monoclonal antibody against IL-1 beta in patients with HF. The results revealed improved HF outcomes in patients with myocardial infarction [2].

The COLCOT trial (COLchicine Cardiovascular Outcomes Trial) randomised patients 30 days after MI, in patients receiving colchicine or placebo. The primary endpoint was a composite of cardiovascular death, resuscitated cardiac arrest, MI, stroke or urgent hospitalisation for angina requiring coronary revascularization. After about 22 months follow up, there was a significant reduction in the incidence of primary endpoint in the group receiving colchicine [19].

A question was raised about the possible anti-inflammatory effects of the major anti-remodelling therapies included in the treatment guideline of HF.

Regarding the ACE-I (angiotensin-converting enzyme inhibitor) and ARB (angiotensin II-receptor blocker), a systematic review and meta-analysis was performed searching for the effect of lowering the proinflammatory cytokines. They included 32 randomised control trials and concluded that ACE-I reduced the levels of CRP, IL6 and TNF alpha, while the ARB were only effective in reducing IL6 [20].

In another study, Dandona et al. observed that ARB inhibits the synthesis of reactive oxygen species and decreases CRP levels by blocking the type 1 receptors, while having anti-inflammatory effects through type 2 receptors [21].

A post hoc analysis of the Valsartan Heart Failure Trial database evaluated the effect of valsartan on CRP levels between patients who received ACE-I at baseline compared to those who did not receive ACE-I at baseline. Valsartan might have a different effect on CRP depending on the prior use of an ACE-I. After a 12-month follow up, valsartan was associated with a reduction of CRP levels in patients who did not receive ACE-I at baseline, but not in those who received ACE-I inhibitors before [7].

The treatment with ACE-I was associated with 2,6-fold reduction of CRP levels in patients with acute ischemic stroke and also a decrease in IL-6 in cases of cardiac surgery [22,23].

Beta-blockers (BB) have been correlated with a significant reduction in TNF alpha in patients with idiopathic dilated cardiomyopathy [24]. The early initiation of beta-blockers in patients with myocardial infarction was associated with a decrease in CRP levels, improving short-term prognosis, and suggesting a beneficial effect on inhibiting cardiac remodelling [25].

Another study reached the conclusion that two of the beta-blockers with proven efficacy in reducing mortality and rehospitalization in HF, have anti-inflammatory and antioxidant effects. Bisoprolol had a greater effect in reducing CRP levels (anti-inflammatory effect), while carvedilol had a more pronounced antioxidant effect (having a more important reduction in reactive oxygen metabolites) [26].

In patients suffering from congestive HF with an EF < 40%, who were randomly assigned to metoprolol or carvedilol, a decrease in CRP concentration was observed [27].

In 333 patients with symptomatic coronary artery disease, beta-blockers use had significantly reduced mean CRP concentrations compared to the patients that did not receive this therapy [28].

Spironolactone was the first MRA (mineralocorticoid receptor antagonist) agent synthetised and used in HF. Miura et al. showed in a study that spironolactone reduced the production of TNF alpha and chemoattractant protein-1 in patients with cardiovascular disease [29]. In another study, haemoglobin A1c levels, which are regarded as an independent risk factor for mortality in patients with chronic HF, were better inhibited by eplerenone (a second generation of MRA) than spironolactone [30].

However, Godfrey et. al, in all four studies performed, did not find any significant effect of spironolactone compared with placebo on CRP levels. This study contests the theory that spironolactone inhibits the progression of HF through anti-inflammatory effects [31].

Ferreira and colleagues concluded that spironolactone use did not influence hsCRP levels after 12 months of treatment for 232 HFpEF patients [32].

Neprilysin Inhibitor–Angiotensin II Receptor Blocker Combination Therapy (Sacubitril/valsartan), also named ARNI, is a novel cardiovascular drug which proved to be very effective for the treatment of congestive HF. It has beneficial effects on endothelial dysfunction, myocardial contraction, hypertension, HF and cardiovascular ischemia-reperfusion injury [33].

ARNI can be regarded as having an anti-inflammatory effect by decreasing the number of T cells. Moreover, ARNI can reduce cardiac fibrosis in ischemic HF cases [34]. CRP reduction was observed in chronic HF patients after 6 months of treatment with ARNI [35].

SGLT2i (Sodium-glucose co-transporter 2 inhibitors) represents a revolutionary discovery in the treatment of HF, with significant reduction in cardiovascular mortality and heart failure hospitalisations. Evidence suggests that SGLT2i have a role in suppressing proinflammatory mechanisms and reducing atherosclerosis progression [36].

In animal subjects, ARNI reduced myocardial fibrosis and improved right ventricular function, while SGLT2i modulated systemic inflammation; moreover, the combination therapy improved vascular density and left ventricle function in animal subjects after myocardial infarction [37].

SGLT2i improve cardiovascular outcomes and reduce cardiac remodelling, but they also have anti-inflammatory effects. In animal subjects, SGLT2i lowered oxidative stress after ischemia-reperfusion and diminished myocardial necrosis and infarct size [38]. Also in animal studies, dapagliflozin, one of the SGLT2i class, led to a significant reduction of collagen synthesis after myocardial infarction, by stimulating anti-inflammatory macrophages and suppressing myofibroblast differentiation [39]. Empagliflozin, another member of the SGLT2 family, can diminish the rate of human fibroblast activation by transforming growth factor β1 in a dose-dependent manner [40]. Empagliflozin lowered renal expression of proinflammatory cytokines (TNF alpha, IL-6) and apoptosis in rats [41].

A meta-analysis which assessed the anti-inflammatory effects of SGLT2i in experimental models underlined that SGLT2i administration led to lower levels of IL-6, CRP and TNF alpha [42]. SGLT2i decrease low grade inflammation, possible by lowering uric acid and insulin levels [43].

A meta-analysis by Wang et al., which included type 2 diabetes patients, indicated lower levels of ferritin, CRP and leptin concentration in the groups receiving SGLT2i therapy versus placebo or standard diabetes treatment [44].

As HF has an enormous prevalence all around the world, it would be daring to be able to define a cardio-inflammatory phenotype, in order to perform an early diagnosis and administer targeted therapy, for reducing the risk of HF development.

There is still an ongoing debate regarding the role of inflammation in HF… is it a cause or a consequence of HF?

## 2. Materials and Methods

### 2.1. Study Hypothesis

The anti-remodelling classes of medication can lower the levels of inflammatory biomarkers (CRP, ESR and fribrinogen) in patients with CHF, irrespective of the EF.

### 2.2. Study Design

The aim of the study is to evaluate the effect of the classic anti-remodelling HF therapies on chronic low-grade systemic inflammation. For this purpose, we evaluated the dynamic of the inflammatory markers (CRP, fibrinogen and ESR) levels between two visits of patients suffering from HF.

The classes of drugs compared were beta blockers (BB), angiotensin I convertase enzyme inhibitors (ACE-I), angiotensin II receptors blockers (ARB), mineralocorticoid antagonists (MRA), the angiotensin II receptors and neprilysin inhibitors (ARNI) and also sodium-glucose co-transporter 2 inhibitors (SGLT2i). 

Enrolment was noted as T0 and the second evaluation T1. 

At first visit (enrolment, T0), we collected data from patients with HF, as follows:

- patients who received a new anti-remodelling agent according to the ESC (European Society of Cardiology) guideline for HF or
- patients who were subject to an up-titration of an already existing anti-remodeling medication according to the ESC guideline for HF. Up-titration was attempted to the maximum tolerated dose.

### 2.3. Study Patients

The study was a retrospective monocentric analysis in which patients with heart failure were included over a period of 3 years: from January 2021 until March 2023. The research focused on patients that received a new class of anti-remodelling therapy at first visit or patients for which the up-titration to the maximum tolerated dose was attempted. The median follow-up of patients was performed after 6 months.

Inclusion criteria:

- patients diagnosed with chronic heart failure who were admitted to hospital for acute decompensated heart failure
- patients with chronic HF that came for the regular medical evaluation.

Exclusion criteria:

- age below 18 years old
- patients with ongoing anti-inflammatory treatment, patients with active systemic inflammatory diseases
- an eGFR < 10 mL/min/1.73 m^2^
- patients receiving anti-inflammatory drugs (non-steroidal or steroidal anti-inflammators, immunosuppressive agents or other medication with established anti-inflammatory effect) for other comorbidities

The primary outcome of the study was to point out if the patients that received higher doses (up until the maximum dose) of a given anti-remodelling HF therapy had a greater reduction in chronic systemic inflammation (evaluated by CRP, fibrinogen, and ESR levels). These higher doses were compared in a 1 versus 1 method to the low and medium doses.

The primary endpoint was a reduction of the inflammatory syndrome, defined as a decrease in the plasmatic level of CRP, fibrinogen or ESR levels at T1 as compared to the T0 value. This was analysed for each particular dose of the active substances from the aforementioned classes. 

The secondary endpoint included a comparison of the inflammatory syndrome reduction, defined as a decrease in the plasmatic level of CRP, fibrinogen or ESR levels from T0 to T1, between the different representatives of each drug class. For beta blockers this included metoprolol succinate (XR/CL form), bisoprolol, carvedilol and nebivolol; for the ACE-I class, we compared perindopril with ramipril and enalapril, including a 4-way comparison (perindopril vs. ramipril vs. enalapril vs. candesartan; candesartan being the only ARB member with already statistically proven significant reduction in cardiovascular mortality).

### 2.4. Ethics Approval and Consent to Participate

All patients have provided written informed consent for data collection and statistical analysis regarding the personal health parameters noted in the medical registries. The study was conducted in accordance with the Declaration of Helsinki and the protocol was approved by the Ethics Committee of “Prof. Dr. Th. Burghele” Clinical Hospital. (Bucharest, Romania; approval no. 3641/2024).

### 2.5. Statistical Analysis

All patient data (demographic, clinical, biological, imagistic) was included in the Microsoft Excel package (2404). Statistical analyses were conducted using SPSS version 26 (SPSS Inc., Chicago, IL, USA). 

As a first step, we calculated frequencies for each medication dose. Also, for the purpose of this study, we computed the arithmetic difference between T1 and T0 scores for C-reactive protein (CRP), erythrocyte sedimentation rate (ESR), and fibrinogen; mean scores for the T0-T1 score differences were calculated for each medication dose. As a second step, we used test–retest analyses with CRP, ESR, and for fibrinogen, as the dependent variables. The analyses were conducted separately for each medication class of interest (i.e., beta-blockers, ACE-I, MRA, ARNI, SGLT-2i), with class representants and the corresponding dosages as the between-subjects variables (only main effects were tested). The significance level (α) of the statistical tests used for the evaluation were considered at a two-tailed probability level of significance of 95% (*p* < 0.05).

## 3. Results

### 3.1. Descriptive Statistics

This retrospective study included 220 patients diagnosed with chronic heart failure, whether admitted to the hospital for an acute episode of decompensation or for a routine bio-clinical evaluation The distribution of patients within the lot, based on EF (reduced ejection fraction, mildly reduced ejection fraction and preserved ejection fraction) was as follows: 111 patients with HFrEF, 23 patients with HFmrEF and 86 patients with HFpEF. Group characteristics are illustrated in Table 1.

All patients were aligned at first visit (T0) to the optimally tolerated guideline-directed medical therapy (OGMT) for HF, whether it meant adding all anti-remodelling therapies (BB, ARNI/ACE-I/ARB, MRA and SGLT2i), according to the EF phenotype, or increasing the dose of the previously mentioned classes up to the maximally tolerated one.

After a median of 6 months (T1), they were re-evaluated with the same parameters, comparing the evolution between T0 and T1. For 123 cases, T1 was a routine follow up visit, while for 84 it was the second HF decompensation. 

The demographic and medical data were recorded from registries. In total, 50.9% of patients were male (112) and 69.1% had an urban background (152); mean age was 70.93 (ranging from 28 to 92 years old).

Taking into consideration the cardiovascular risk factors, the average body mass index (BMI) was 26.01, with a mean value of 29 (1 of the patients being underweight, 14 having a normal body mass index, 72 being overweight, 54 suffering from I degree obesity, 14 with II degree obesity and 2 with III degree obesity). A total of 49 of patients were smokers (32.3%), with 11 of them being heavy smokers, defined as above 20 pack-years (5.1%). A small number from the group admitted chronic alcohol consumption (27, 13.3%), with 26 of them accounting for heavy scores (above 3 units of alcohol/day). 

The initial group consisted of a total of 220 patients with HF, including all three phenotypes of EF. The patient’s distribution by EF (reduced ejection fraction, mildly reduced ejection fraction and preserved ejection fraction) was HFrEF (N = 111), HFmrEF (N = 23) and HFpEF (N = 86).

Based upon parameters indicating non-normality of data distribution (skewness, kurtosis, and tests for normality of data) and by identifying a significant correlation with urinary markers, indicative of an active urinary infection (e.g., leukocyturia, hematuria, and leukocyte cylinders), the decision was made to exclude patients with values higher than three standard deviations. Based on this criterion, 13 patients from the whole group of 220 were excluded from further analyses resulting in a total N = 207 participants. 

### 3.2. Analytic Statistics

Our research had the purpose to demonstrate if there was a difference in inflammatory markers (CRP, ESR and fibrinogen) depending on the classes of anti-remodelling medication that the patients received (classes evaluated: beta-blockers, MRA, ACE-I, ARNI, SGLT2i, and candesartan from ARB class). 

The first step was to count the frequency of each active substance received at first evaluation (T0) by the 207 patients. At the same time, we analysed the mean values for the difference between T0 and T1 (the second evaluation) in CRP, ESR and fibrinogen, for the population that received each class of medication (data are presented in Table 2).

To evaluate if a certain drug dose influenced in a greater manner the dynamics of inflammatory markers between T0 and T1, test–retest analyses were performed for each class of anti-remodelling therapy.

For beta-blockers, three sets of test–retest types of analyses were conducted testing each dose from the three beta-blockers the patients received as a group (carvedilol, metoprolol, bisoprolol), with the dependent variables being CRP, ESR and fibrinogen. 

**Figure 1:**
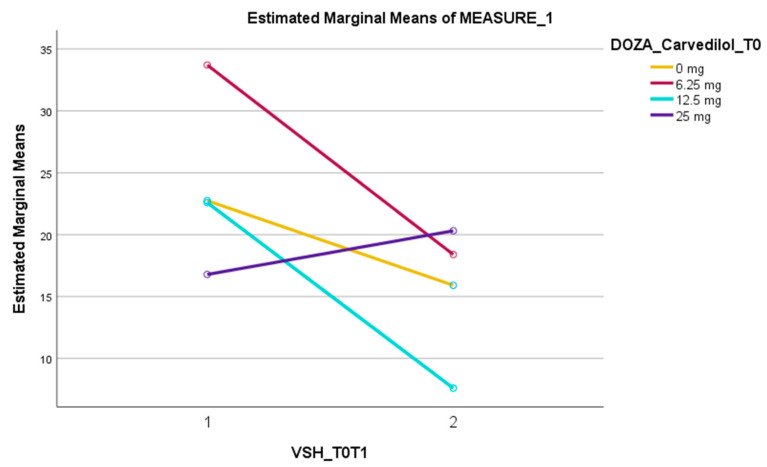
The dynamics of ESR from T0 to T1 in patients who did not receive carvedilol vs. those with 6.25 mg/12.5 mg/25 mg bi daily of carvedilol.

Regarding the dynamics of ESR, there were statistically significant differences in the association with the dose of Carvedilol, with Wilk’s Lambda F (3,144) = 2.82, *p* < 0.05, and η_p_^2^ = 0.053. As can be seen in the table with average values (Table 3 and Table 4) and in Figure 1, the dynamics of ESR were different for each dose of Carvedilol: at the low (6.25 mg, bi daily) dose, the average ESR values decreased from 33.70 mm/h to 18.39 mm/h; for the 12.5 mg dose, the mean ESR values decreased the most, from 22.58 mm/h to 7.59 mm/h, while for the 25 mg dose, the mean ESR values increased slightly, from 16.78 mm/h to 20.31 mm/h.

There were no significant differences after beta-blockers administration in the CRP or fibrinogen dynamics (no significant *p* value), as shown in Table 3.

In the case of ACE-I, because there were only a few patients who received enalapril (N = 5, as seen in Table 1), we proceeded at evaluating test–retest analysis only for perindopril and ramipril. The separate tests with the three dependent variables (CRP, ESR and fibrinogen) showed no significant differences between the 2.5, 5 and 10 mg doses, as can be seen in Table 5.

Because the ACE-I and ARB classes both inhibit the same neurohormonal system (renin-angiotensin-aldosterone), which is activated in CHF, and because they are often compared side by side for their effect, we also analysed the ARB. From this class we tested only the patients receiving candesartan. The tests showed no statistical significance in the difference of the inflammatory markers’ dynamics, whether the patients had received 4 mg, 8 mg, 16 mg or 32 mg of candesartan. The *t*-test results are listed below (Table 6).

From the MRA class, we evaluated in our study patients who received spironolactone. As can be seen in Table 7, there was no statistical significance in the influence of spironolactone on the dependent variables (CRP, ESR and fibrinogen).

Regarding ARNI doses, significant results were obtained in association with the dynamics of CRP values, with Wilk’s Lambda F(3,203) = 4.48, *p* < 0.005, and η_p_^2^ = 0.062; the data can be seen in Table 8 and Table 9. It is worth mentioning that this significant result is enhanced by the fact that there were also significant differences in the CRP value at T0, as those receiving higher doses of ARNI had significantly higher CRP values at T0 (F(1,203) = 5.24, *p* < 0.05, η_p_^2^ = 0.025). 

**Figure 2:**
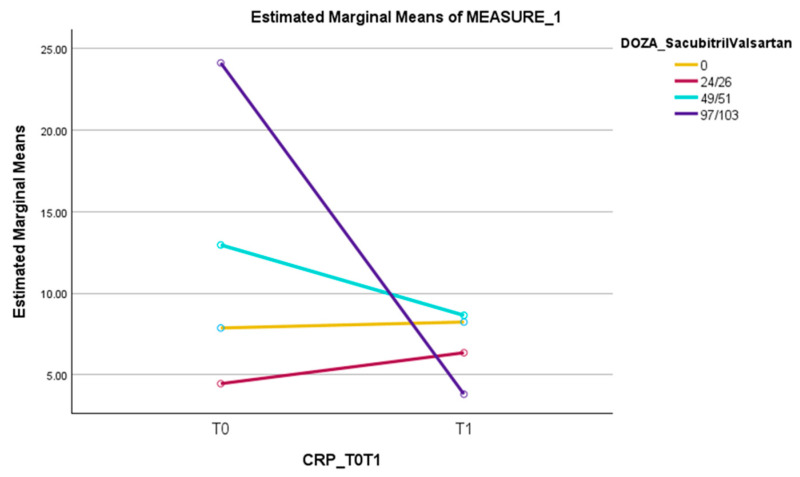
The dynamics of CRP from T0 to T1 in patients who did not receive sacubitril/valsartan versus those with 24/26 mg bi daily, 49/51 mg bi daily or 97/103 mg bi daily of sacubitril/valsartan.

The average values of CRP increased slightly for those without ARNI from 7.86 mg/L to 8.23 mg/L; those on the 24/26 mg dose bi daily increased from 4.45 mg/L to 6.35 mg/L; for the patients taking the dose of 49/51 mg bi daily, the CRP levels decreased from 12.96 mg/L to 8.64 mg/L; and in those with the 97/103 dose bi daily, the average CRP values decreased from 24.12 mg/L to 3.80 mg/L. The results for each group of patients are presented in the table above (Table 9 and in Figure 2).

However, there was no difference between T1 and T0 in ESR or fibrinogen dynamics, in the patients receiving different doses of ARNI; the data is available in Table 8.

The last class of anti-remodelling active substances evaluated were SGLT2i, with separate test–retests analysis of empagliflozin and dapagliflozin. No statistical significance was observed in the dynamics of CRP, ESR or fibrinogen. Results are displayed in Table 10.

## 4. Discussion

In our study, we tried to evaluate a much studied and still debatable field of research. We searched for the anti-inflammatory effect of anti-remodelling classes used in the treatment of CHF.

Our retrospective study included and collected data at enrolment (T0) from patients with HF that had an adjustment in their treatment between T0 and T1. In this way, patients received a new class of anti-remodelling medication according to the ESC guideline of HF or the patients that already had that class in their treatment were subjected in an up-titration of the dose. Our research evaluated the effects of beta-blockers, ACE-I, ARB, MRA, SGLT2i and ARNI on three routinely used markers of non-specific systemic inflammation (CRP, ESR and fibrinogen) in clinical practice. We compared different doses of each active substance from those classes of medication, and then proceeded to compare intra-class, between active substances, the effects on dynamics of inflammatory biomarkers. In our study we did not compare the magnitude of effect between the phenotypes of HF (HFrEF, HFmrEF or HFpEF).

From beta-blockers, carvedilol reached significance in reducing the ESR level from T0 to T1 (*p* = 0.049). The dynamics of ESR were different for each dose of carvedilol: at the low dose, the average ESR values decreased from 33.70 mm/h to 18.39 mm/h; and for the 12.5 mg dose, the mean ESR values decreased the most, from 22.58 mm/h to 7.59 mm/h. However, for the 25 mg dose, the mean ESR values increased slightly, from 16.78 mm/h to 20.31 mm/h.

In the case of CRP and fibrinogen, there was no difference in reduction between carvedilol doses.

For the other beta-blockers, bisoprolol and metoprolol, no significant result was obtained.

In the case of ACE-I, ARB, MRA or SGLT2i, the results did not reach statistical significance in the dynamics for any of the CRP, ESR or fibrinogen levels.

For patients receiving ARNI, a significant result was observed in the dynamics of CRP (*p* = 0.004). The effect was different depending on the dose of ARNI: with a decrease in CRP values for the 49/51 mg and 97/103 mg dose, the greatest reduction being reached in the high dose of ARNI. While for the 24/26 mg dose and for the group of patients that did not receive ARNI, a slight increase in CRP levels was seen.

A meta-analysis and systematic review evaluated the lowering effect of ACE-I and ARB on three proinflammatory cytokines: CRP, IL6 and TNF alpha. They included 32 randomised control trials and concluded that ACE-I significantly reduced the CRP ([95% CI]; −1.17 mg/L to −0.33 mg/L, *p* = 0.002), while ARB did not affect the CRP levels [20].

In another study, Dandona et al., showed that ARB decreased the CRP [21].

In our study, in which 91 patients received ACE-I (51-perindopril, and 37 ramipril) and 34 patients were treated with candesartan, none of these active substances proved to significantly reduce CRP, ESR or fibrinogen.

In a subanalysis of the BRIGHT-D trial, two beta-blockers, bisoprolol and carvedilol, reduced the hsCRP level, with the first one having a greater effect (from 3.35 ng/mL to 2.69 ng/mL, *p* = 0.001) than the later one (from 3.38 ng/mL to 2.85 ng/mL, *p* = 0.047) [26]. The same conclusion was obtained also, by Nagatomo et. al, in another study [27].

Jenkins et. al, demonstrated a reduction in the CRP level in patients with HF of ischemic etiology that received BB, in comparison to those who did not (−1.2 mg/L mean CRP level for BB group, *p* < 0.001) [28].

In our research, in which 170 patients had in their treatment BB (115-metoprolol succinate, 18 bisoprolol, and 37 carvedilol), no BB influenced the CRP and fibrinogen levels.

However, carvedilol reached a significant reduction in ESR levels from T0 to T1 (*p* = 0.049), an effect observed in the low (6.25 mg, bi daily) and medium (12.5 mg, bi daily) doses.

In four separate studies, Godfrey et al., found no significant effect of spironolactone on the CRP levels [31]. This result was consistent with our results. Spironolactone, which was the only MRA studied (received by 104 patients) failed to prove a reduction in CRP, ESR or fibrinogen.

A study conducted by Goncalves et al., of patients with HF that received ARNI, demonstrated a significant reduction in CRP levels (from to 2.5 mg/L to 2.2 mg/L, *p* = 0.014) [35]. 

Also, in our study, patients who received ARNI (N = 30) had a statistically significant decrease in CRP levels from baseline (*p* = 0.004). This result was obtained in the medium (49/51 mg; N = 17 patients) and high (97/103 mg; N = 7 patients) dose groups of patients, while for the low (24/26 mg; N = 6 patients) dose, an opposite effect of slight increase in CRP was observed.

Regarding the SGLT2i class, a meta-analysis which evaluated the anti-inflammatory effect of SGLT2i on CRP in experimental models, showed a reduction in CRP levels (−2.17 mg/L mean reduction; [CI 95%; −2.80 mg/L to −1.53 mg/L]) [42].

A reduction in CRP value was observed also by Wang et. al, in a study on diabetes mellitus type 2 patients, who received SGLT2i [44].

In our study, 33 patients had as part of their treatment an SGLT2i (31-dapagliflozin and 2 empagliflozin). But no significant decrease in the dynamic of CRP, ESR or fibrinogen was noted between T0 and T1.

### Limitations

The study was a retrospective observational analysis of a group of patients. Although it gathered a heterogeneous profile of patients, having no randomization may have lowered the statistical strength of the results.

The value of inflammatory markers could have been negatively influenced by the fact that for 84 patients, the second evaluation (T1) was in the course of an acute decompensation of CHF, while for the other 123 it was a routine evaluation. To avoid a false increase in inflammatory biomarkers, we excluded from the beginning patients who had at the moment of testing an infection, condition pointed out by leukocytosis, leukocyturia or a positive uroculture.

## 5. Conclusions

The anti-remodelling medications are the only substances that can have an impact on the survival of patients diagnosed with HF. They also improve the prognosis and morbidity, by decreasing the rehospitalization rate [45]. The mechanisms that generate these effects are in a great amount demonstrated and known, especially for the classic anti-remodelling therapies for HF (beta-blockers, ACE-I, ARB and MRA) [46].

The SGLT2i and ARNI emerged as new classes which showed further improvement in the prognosis of patients (both by reducing mortality rates and rehospitalization rates of patients) that were already treated with the aforementioned medications [47].

HF is accompanied by a low-grade chronic and systemic inflammation, a phenomenon which still raises questions of causality and effect (is the inflammation the physiopathological process which generates and induces progression of HF, or is it only a consequence of ischemia, hypertension, valvulopathies, cardiomyopathy or other cardiac inflicting processes?) [48,49].

Our research showed a significant reduction in ESR levels from T0 to T1 for the patients receiving carvedilol (*p* = 0.049). This effect was achieved in the low and medium dose (6.25 mg bi daily and 12.5 mg bi daily), while for the high dose (25 mg bi daily), the ESR slightly increased between evaluations. For the other beta-blockers (bisoprolol and metoprolol), no significant result was obtained. 

In the case of ACE-I, ARB, MRA or SGLT2i, the results did not reach statistical significance in the dynamics for any of the CRP, ESR or fibrinogen levels.

On the other hand, a statistically significant result was observed in patients receiving ARNI, regarding the dynamics of CRP (*p* = 0.004). The effect was different depending on the dose of ARNI: a decrease in CRP values was observed when administering the medium (49/51 mg, bi daily) and high (97/103 mg, bi daily) dose, with the greatest reduction in CRP values being reached in the case of a high dose of ARNI treatment. While for the patients who received the 24/26 mg dose bi daily and for the group of patients that did not receive ARNI at all, a slight increase in CRP levels was seen.

Although these established classes of medication for the treatment of heart failure have proven anti-remodelling effects, with direct clinical implications on mortality and hospitalisation rates, their anti-inflammatory role is still waiting to be proven. 

Future research efforts relying on similar studies, but with a larger scale of enrolled patients, are prone to deliver a definite verdict, which can improve the pharmacodynamic deciphering of these classes.

## Figures and Tables

**Table 1:** Descriptive statistics of the entire group of patients (N = 220).

	HFrEF (N = 111)	HFmrEF (N = 23)	HFpEF (N = 86)
**Descriptives**			
Gender			
Male	72	15	25
Female	39	8	61
Age (mean/min-max)	68.2 (28–92)	69.83 (47–91)	74.62 (41–92)
**Comorbidities**			
Blood Pressure (mean)			
Systolic	130.40 (90–230)	140.00 (100–180)	135.87 (80–190)
Diastolic	78.53 (50–134)	81.91 (60–100)	76.35 (50–105)
Chronic coronary syndrome	92 (82.9%)	11 (47.8%)	59 (68.6%)
Acute coronary syndrome	52 (46.8%)	4 (17.4%)	12 (14.0%)
Acute myocardial infarction	53 (47.7%)	5 (21.7%)	10 (11.6%)
Dilated cardiomyopathy	53 (47.7%)	3 (13.0%)	3 (3.5%)
Hypertrophic cardiomyopathy	3 (2.7%)	1 (4.3%)	12 (14.0%)
Restrictive cardiomyopathy	6 (5.4%)	--	1 (1.2%)
Atrial fibrillation	50 (45.0%)	17 (73.9%)	45 (52.3%)
Atrioventricular block			
Grade 1	101 (91.0%)	21 (91.3%)	82 (95.3%)
Grade 2	9 (8.1%)	1 (4.3%)	3 (3.5%)
Grade 3	1 (0.9%)	1 (4.3%)	--
Diabetes mellitus	51 (45.9%)	13 (56.5%)	32 (37.2%)
Dyslipidemia	96 (86.5%)	17 (73.9%)	79 (91.9%)
Atheromatosis	67 (60.4%)	8 (34.8%)	43 (50.0%)
Cerebrovascular events	5 (4.5%)	--	10 (11.6%)
Acute peripheral ischemia	11 (9.9%)	--	2 (2.3%)
Chronic Obstructive Pulmonary Disorder	25 (22.5%)	3 (13.0%)	10 (11.6%)
Chronic kidney disease	77 (69.4%)	17 (73.9%)	47 (54.7%)
**Symptoms**			
Dyspnea (class)			
NYHA 1	3 (3.7%)	1 (4.3%)	8 (9.3%)
NYHA 2	34 (30.6%)	12 (52.2%)	54 (62.8%)
NYHA 3	58 (52.3%)	10 (43.5%)	21 (24.4%)
NYHA 4	16 (14.4%)	--	3 (3.5%)
Cough	26 (23.4%)	2 (8.7%)	14 (16.3%)
Astheny/fatigability	92 (82.9%)	16 (69.6%)	65 (75.6%)
Angina	62 (55.9%)	7 (30.4%)	45 (52.3%)
Palpitations	88 (79.3%)	15 (65.2%)	64 (74.4%)
Syncope	7 (6.3%)	1 (4.3%)	11 (12.8%)
Jugular vein distension	42 (37.8%)	4 (17.4%)	10 (11.6%)
Ventricular gallop (III sound)	16 (17.2%)	4 (17.4%)	6 (7.4%)
Oedema	61 (55.0%)	6 (26.1%)	24 (27.9%)
Anasarca	15 (13.5%)	--	1 (1.2%)

**Table 2:** Frequencies of each active substance use in the group of 207 patients. Mean values of the difference in inflammatory markers (CRP, ESR and fibrinogen) between T0 and T1.

Medication	N	% from Total N (207)	T0-T1 (Mean Scores)		
			CRP mg/L	ESR mm/h	Fibrinogen mg/dL
**Beta-blockers**					
Metoprolol (dose per day)	115	55.6%	−1.07	−1.12	−20.47
50 mg	41	19.8%	−2.74	−4.46	−21.31
100 mg	49	23.7%	−0.12	2.78	−19.00
150 mg	10	4.8%	−3.56	−1.83	2.80
200 mg	15	7.2%	2.03	−3.16	−48.20
Bisoprolol (dose per day)	18	8.7%	−1.48	−0.61	−7.10
2.5 mg	4	1.9%	1.98	8.25	61.33
5 mg	11	5.3%	−3.54	−1.50	−39.16
10 mg	3	1.4%	1.45	−29.00	−20.00
Carvedilol (bi daily)	37	17.87%	−0.54	2.18	23.90
6.25 mg	7	3.4%	5.70	−5.25	52.66
12.5 mg	16	7.7%	−8.14	−7.30	−13.00
25 mg	14	6.8%	5.031	11.21	38.25
**ACE-I**					
Perindopril (dose per day)	54	26.1%	−0.49	1.43	−1.35
2.5 mg	2	1.0%	0.66	0.00	−2.00
5 mg	29	14.0%	−3.76	−1.72	−13.07
10 mg	23	11.1%	3.86	5.60	25.66
Ramipril (bi daily)	37	17.4%	−3.32	2.00	−15.11
2.5 mg	11	5.3%	−8.07	−7.66	−3.50
5 mg	15	7.2%	−1.36	5.54	12.85
10 mg	11	5.3%	−1.09	5.30	−68.20
Enalapril (bi daily)					
10 mg	5	2.4%	−9.39	−11.66	−35.50
**ARB** (total dose per day)					
Candesartan	34	16.4%	4.05	−2.23	−0.13
4 mg	0	0%	-	-	-
8 mg	13	6.3%	4.64	−5.37	−52.00
16 mg	10	4.8%	6.07	−4.62	−36.00
32 mg	11	5.3%	1.51	3.00	−8.00
**MRA** (total dose per day)					
Spironolactone	104	50.2%	−1.15	−1.67	−1.41
25 mg	78	37.7%	−0.69	3.52	7.68
50 mg	24	11.6%	−2.60	−7.18	−45.00
**ARNI (bi daily dose)**					
Sacubitril/valsartan	30	14.4%	−5.63	−1.76	14.00
24/26 mg	6	2.9%	1.89	−13.00	−44.00
49/51 mg	17	8.2%	−4.31	0.81	4.75
97/103 mg	7	3.4%	−20.31	−4.20	−9.00
**SGLT2i (once daily)**					
Dapagliflozin 10 mg	31	15.0%	−4.91	−0.61	−32.47
Empagliflozin 10 mg	2	1.0%	−0.58	−0.10	−4.37

**Table 3:** *t*-tests results for the effect of beta-blockers (Carvedilol, Metoprolol and Bisoprolol) on the dynamics of CRP, ESR and fibrinogen.

Effect	F	Hypothesis df	Error df	Sig.	Partial Eta Squared
ESR T0–T1	1.469	1.000	144.000	0.228	0.010
ESR T0–T1 * Carvedilol dose	**2.682**	**3.000**	**144.000**	**0.049**	**0.053**
ESR T0–T1 * Metoprolol dose	0.823	4.000	144.000	0.513	0.022
ESR T0–T1 * Bisoprolol dose	1.407	3.000	144.000	0.243	0.028
CRP T0-T1	0.120	1.000	196.000	0.729	0.001
CRP T0–T1 * Carvedilol dose	2.240	3.000	196.000	0.085	0.033
CRP T0–T1 * Metoprolol dose	0.486	4.000	196.000	0.746	0.010
CRP T0–T1 * Bisoprolol dose	0.240	3.000	196.000	0.868	0.004
Fibrinogen T0-T1	0.521	1.000	85.000	0.472	0.006
Fibrinogen T0–T1 * Carvedilol dose	0.465	3.000	85.000	0.707	0.016
Fibrinogen T0–T1 * Metoprolol dose	0.717	4.000	85.000	0.583	0.033
Fibrinogen T0–T1 * Bisoprolol dose	0.760	3.000	85.000	0.520	0.026

**Table 4:** ESR dynamics from T0 to T1 in the patients receiving Carvedilol.

Carvedilol Dose	ESR T0–T1	Mean	Std. Error	95% Confidence Interval	
				Lower Bound	Upper Bound
Without Carvedilol	T0	22.746	6.582	9.735	35.756
	T1	15.910	6.409	3.242	28.578
**6.25 mg**	**T0**	**33.709**	**12.447**	**9.107**	**58.311**
	**T1**	**18.394**	**12.119**	**−5.561**	**42.348**
**12.5 mg**	**T0**	**22.583**	**10.362**	**2.101**	**43.065**
	**T1**	**7.598**	**10.089**	**−12.345**	**27.540**
25 mg	T0	16.783	9.787	−2.561	36.127
	T1	20.312	9.529	1.477	39.147

**Table 5:** *t*-tests results for the effect of ACE-I (perindopril and ramipril) on the dynamics of CRP, ESR and fibrinogen.

Effect	F	Hypothesis df	Error df	Sig.	Partial Eta Squared
CRP T0–T1	0.449	1.000	200.000	0.503	0.002
CRP T0–T1 * perindopril dose	1.020	3.000	200.000	0.385	0.015
CRP T0–T1 * ramipril dose	0.894	3.000	200.000	0.445	0.013
ESR T0-T1	0.365	1.000	148.000	0.547	0.002
ESR T0–T1 * perindopril dose	0.986	3.000	148.000	0.401	0.020
ESR T0–T1 * ramipril dose	1.268	3.000	148.000	0.287	0.025
Fibrinogen T0–T1	0.125	1.000	89.000	0.725	0.001
Fibrinogen T0–T1 * perindopril dose	0.274	3.000	89.000	0.844	0.009
Fibrinogen T0–T1 * ramipril dose	0.695	3.000	89.000	0.557	0.023

**Table 6:** *t*-tests results for the effect of candesartan on the dynamics of CRP, ESR and fibrinogen.

Effect	Value	F	Hypothesis df	Error df	Sig.	Partial Eta Squared
CRP T0–T1	0.992	1.687	1.000	203.000	0.196	0.008
CRP T0–T1 * candesartan dose	0.980	1.402	3.000	203.000	0.243	0.020
ESR T0–T1	0.998	0.367	1.000	151.000	0.545	0.002
ESR T0–T1 * candesartan dose	0.010	0.521	3.000	151.000	0.668	0.010
Fibrinogen T0–T1	0.987	1.192	1.000	92.000	0.278	0.013
Fibrinogen T0–T1 * candesartan dose	0.984	0.511	3.000	92.000	0.676	0.016

**Table 7:** *t*-tests results for the effect of spironolactone on the dynamics of CRP, ESR and fibrinogen.

Effect	F	Hypothesis df	Error df	Sig.	Partial Eta Squared
CRP T0–T1	0.786	1.000	204.000	0.376	0.004
CRP T0–T1 * spironolactone dose	0.221	2.000	204.000	0.802	0.002
ESR T0–T1	0.764	1.000	152.000	0.383	0.005
ESR T0–T1 * spironolactone dose	2.499	2.000	152.000	0.086	0.032
Fibrinogen T0–T1	1.250	1.000	93.000	0.266	0.013
Fibrinogen T0–T1 * spironolactone dose	1.418	2.000	93.000	0.247	0.030

**Table 8:** *t*-tests results for the effect of ARNI on the dynamics of CRP.

Effect	F	Hypothesis df	Error df	Sig.	Partial Eta Squared
CRP T0–T1	5.249	1.000	203.000	0.023	0.025
CRP T0–T1 * sacubitril/valsartan dose	**4.488**	**3.000**	**203.000**	**0.004**	**0.062**
ESR T0–T1	1.492	1.000	151.000	0.224	0.010
ESR T0–T1 * sacubitril/valsartan dose	0.848	3.000	151.000	0.470	0.017
Fibrinogen T0–T1	0.340	1.000	92.000	0.561	0.004
Fibrinogen T0–T1 * sacubitril/valsartan dose	0.183	3.000	92.000	0.908	0.006

**Table 9:** CRP dynamics from T0 to T1 in the patients receiving ARNI.

Sacubitril/Valsartan Dose	CRP_T0T1	Mean	Std. Error	95% Confidence Interval	
				Lower Bound	Upper Bound
Without sacubitril/valsartan	T0	7.869	0.946	6.003	9.735
	T1	8.233	0.821	6.614	9.853
24/26 mg bi daily	T0	4.454	5.647	−6.680	15.588
	T1	6.352	4.901	−3.311	16.015
**49/51 mg** bi daily	**T0**	**12.960**	**3.063**	**6.922**	**18.998**
	**T1**	**8.645**	**2.658**	**3.404**	**13.885**
**97/103 mg** bi daily	**T0**	**24.121**	**4.773**	**14.711**	**33.532**
	**T1**	**3.809**	**4.142**	**−4.358**	**11.975**

**Table 10:** *t*-tests results for the effect of SGLT2i on the dynamics of CRP, ESR and fibrinogen.

Effect	F	Hypothesis df	Error df	Sig.	Partial Eta Squared
CRP T0–T1	1.871	1.000	204.000	0.173	0.009
CRP T0–T1 * dapagliflozin 10 mg	2.805	1.000	204.000	0.096	0.014
CRP T0–T1 * empagliflozin 10 mg	0.956	1.000	204.000	0.329	0.005
VSH T0–T1	0.124	1.000	152.000	0.726	0.001
VSH T0–T1 * dapagliflozin 10 mg	0.021	1.000	152.000	0.886	0.000
VSH T0–T1 * empagliflozin 10 mg	0.156	1.000	152.000	0.694	0.001
Fibrinogen T0–T1	1.356	1.000	94.000	0.247	0.014
Fibrinogen T0–T1 * dapagliflozin 10 mg	2.088	1.000	94.000	0.152	0.022
Fibrinogen T0–T1 * empagliflozin 10 mg		0.000	94.000		

## Data Availability

The original contributions presented in the study are included in the article, further inquiries can be directed to the corresponding author/s.
